# Global Longitudinal Strain and Brain Natriuretic Peptide as Prognostic Biomarkers for Asymptomatic Severe Aortic Regurgitation With Preserved Ejection Fraction: Protocol for a Systematic Review and Meta-Analysis

**DOI:** 10.2196/71380

**Published:** 2026-08-07

**Authors:** Myung-Rho Kim, Taha Shaikh, Spencer Taylor, Shawn Wang, Vidhani Goel, Michael DiCaro, Banveet Kaur Khetarpal, Kavita Batra

**Affiliations:** 1 Department of Internal Medicine, Kirk Kerkorian School of Medicine at UNLV, Las Vegas, NV, United States; 2 Department of Epidemiology and Biostatistics, School of Public Health, University of Nevada, Las Vegas, Las Vegas, NV, United States; 3 Division of Cardiovascular Medicine, Department of Internal Medicine, Kirk Kerkorian School of Medicine at UNLV, Las Vegas, NV, United States; 4 Office of Research and Department of Medical Education, Kirk Kerkorian School of Medicine at UNLV, 625 Shadow Ln, Las Vegas, NV, 89106, United States, 1 (702) 895-3011

**Keywords:** brain natriuretic peptide, BNP, global longitudinal strain, GLS, aortic regurgitation, AR, preserved ejection fraction, EF, prognostic biomarkers, risk stratification, asymptomatic severe AR, meta-analysis, systematic review, left ventricular ejection fraction, LVEF, cost-effectiveness

## Abstract

**Background:**

Brain natriuretic peptide (BNP) and global longitudinal strain (GLS) are emerging biomarkers used to risk-stratify patients with asymptomatic severe aortic regurgitation (AR) and preserved ejection fraction (EF). Although numerous clinical trials have investigated the efficacy of these biomarkers in patients with aortic stenosis, only a limited number have examined these biomarkers in patients with AR. Therefore, the proposed systematic review and meta-analysis seeks to assess the prognostic value of BNP and/or GLS in patients with severe asymptomatic AR and preserved EF.

**Objective:**

This is a protocol for a systematic review and meta-analysis that will aggregate and synthesize high-quality clinical data on the usefulness of BNP and GLS as prognostic indicators for asymptomatic severe AR with preserved EF. By providing a comprehensive review, our study will have a significant impact in determining surgical candidacy in this patient population.

**Methods:**

In accordance with the PRISMA-S (Preferred Reporting Items for Systematic reviews and Meta-Analyses literature search extension), which is an extension of the PRISMA (Preferred Reporting Items for Systematic Reviews and Meta-Analyses) statement for reporting literature searches in systematic reviews, a comprehensive search of databases, including PubMed, Cochrane, and Embase, will be performed to retrieve peer-reviewed, English-language, observational, and experimental studies published from inception to November 2024. Studies that investigated patients aged ≥18 years with severe AR and preserved EF will be included. The National Heart, Lung, and Blood Institute tool will be used to assess the quality of the studies.

**Results:**

Search strategy development for this systematic review began in November 2024. A Peer Review of Electronic Search Strategies review of the search strategy with 2 academic librarians occurred in December 2024, and the final search strategy was finalized by the team of investigators in January 2025. Database queries and screening of studies began in January 2025 with title screening, followed by abstract screening in January and February 2025. Full-text screening took place from February to April 2025. Data extraction occurred between April and May 2025. Synthesis and risk of bias assessment occurred between April and May 2026, followed by data analysis between June and July 2026. Manuscript drafting will begin between June 2026 and July 2026**,** with manuscript writing and data dissemination continuing from May 2026 to August 2026. Findings will be submitted to a peer-reviewed journal by August 2026.

**Conclusions:**

This systematic review will synthesize the existing evidence to determine the prognostic value of BNP and GLS in patients with asymptomatic severe AR and preserved EF, which could inform future clinical guidelines for the management of this population.

## Introduction

Approximately 4.9% of the general population is affected by aortic regurgitation (AR) [1], and patients with severe AR have elevated rates of mortality. Among patients with severe AR, aortic valve surgery is recommended for symptomatic patients as a Class I indication irrespective of left ventricular (LV) systolic dysfunction [2-4]. In asymptomatic patients, LV systolic dysfunction is a critical variable in determining surgical candidacy because severe AR with LV systolic dysfunction is associated with higher mortality rates [3,5-7]. Therefore, regular follow-up with routine echocardiograms is recommended during the asymptomatic period for monitoring [8,9].

Recent studies suggest that brain natriuretic peptide (BNP) and global longitudinal strain (GLS) are emerging biomarkers for risk stratification in patients with asymptomatic severe AR and preserved ejection fraction (EF). These 2 biomarkers are believed to be superior to LV EF because they can detect subclinical myocardial damage before LV dysfunction occurs [10-12]. BNP is a known marker for diagnosing and monitoring heart failure. However, because BNP responds to cardiac wall stress from volume or pressure overload, it can also detect subclinical LV dysfunction in patients with aortic valve disease [13]. GLS is a parameter derived from speckle-tracking echocardiography that is easy to calculate and is helpful for identifying subclinical myocardial dysfunction. GLS measures longitudinal shortening of the LV, and it is mainly used for the quantification of LV function. However, GLS is more sensitive than the EF in assessing early LV dysfunction in patients with heart failure [14-18]. If BNP and GLS can identify myocardial damage before LV dysfunction develops, then these 2 biomarkers may serve as better prognostic indicators than LVEF in patients with asymptomatic AR. Although many clinical trials have examined the efficacy of these biomarkers in patients with aortic stenosis, there are few trials examining these biomarkers in patients with AR. Therefore, the role of BNP and GLS as prognostic markers in asymptomatic severe AR requires further investigation [19,20].

Both BNP and GLS are easy to measure at low cost, and if these 2 biomarkers are demonstrated to be superior to LV EF for risk stratification in patients with asymptomatic severe AR and preserved EF, this research could significantly improve the outcomes for these patient populations and alter the practicality of routine measurement.

Currently, there is no meta-analysis investigating BNP as a prognostic marker in asymptomatic severe AR with preserved EF, and there are only 2 meta-analyses using GLS. The meta-analysis published in 2020 included 6 studies, but substantially more data are now available [21]. The second meta-analysis, published in 2024, combined both aortic stenosis and AR [22]. Considering the lack of a meta-analysis evaluating BNP and the need for an updated GLS investigation, this meta-analysis will provide a comprehensive review of these biomarkers as prognostic indicators in asymptomatic severe AR with preserved EF. The primary end point will be symptom development (ie, new-onset dyspnea, angina, or syncope attributable to AR). Secondary end points will include the emergence of LV dysfunction (defined as a decline in LVEF below 50%), any indication for surgical intervention, the occurrence of major adverse cardiac events (MACEs), and all-cause mortality. Where multiple time points are reported, we will extract data from the longest available follow-up and conduct sensitivity analyses by time point where feasible.

## Methods

### Ethical Considerations

International review board approval was not required for this systematic review, as it did not involve any patient interaction and was conducted solely using publicly available published data. To ensure project integrity, the review was registered with PROSPERO (CRD42024579540) on August 24, 2024. PROSPERO is an international database with which systematic reviews on a variety of topics, including health care, can register to prevent duplication and reduce reporting bias by comparing the protocol with the final review.

### Review Questions

Guided by the population, exposure, control or comparison, outcome, and study design framework [23], we aim to answer the following question: “What is the prognostic value of BNP and/or GLS in asymptomatic severe AR cases with preserved ejection fraction?”

### Inclusion and Exclusion Criteria

The systematic review used the population, exposure, control or comparison, outcome, and study design method as detailed in (Table 1).

The systematic review included case studies, randomized controlled trials (RCTs), and single-arm studies. The meta-analysis focused exclusively on RCTs and relevant follow-up studies. Only English-language papers published before November 28, 2024, were included. Case reports, abstract-only papers, animal studies, commentaries, position papers, opinions, and editorials were excluded. Rival meta-analyses and systematic reviews were also excluded.

The study population consisted of patients aged >18 years with asymptomatic severe AR of the native heart valves who had preserved LV EF and normal LV diastolic and systolic diameters as defined by the European Society of Cardiology. Patients who did not meet these criteria, including those aged <18 years, with mild to moderate AR, reduced LV EF, previous transcatheter or surgical aortic valve replacement, or symptomatic AR, were excluded from the systematic review. Studies meeting the population inclusion criteria that used GLS and/or BNP as markers of progression were included in the study. Studies that did not include these measurements or the outcomes of interest were excluded.

**Table 1.** Inclusion and exclusion criteria guided by the population, exposure, control or comparator, outcomes, and study design (PECOS) framework.

PECOS elements	Inclusion criteria	Exclusion criteria
Population	Adults aged ≥18 years with asymptomatic severe aortic regurgitation with normal EF^a^	Patients aged <18 years with symptomatic mild to moderate aortic regurgitation, reduced EF, or a previous history of surgical or transcatheter aortic valve replacement
Exposure	Severe aortic regurgitation	N/A^b^
Comparator	Severe aortic regurgitation managed under standard clinical surveillance	N/A
Outcome	Primary end point: symptomatic changes, left ventricular function changes, or any additional indications requiring surgical interventions. Secondary end point: major adverse cardiac events or all-cause mortality	N/A
Study design	All observational and experimental studies published in English	Case reports, conference abstracts, previous systematic meta-analyses, letters to the editor, short commentaries, documentaries, non–English-language papers, and studies without relevant data points

a EF: ejection fraction.

b N/A: not available.

### Information Sources and Search Strategy

Bibliographic databases, including PubMed, Cochrane, and Embase, were used from database inception to November 28, 2024. The search strategy was developed by a librarian with expertise in medical sciences and was designed in accordance with the Peer Review of Electronic Search Strategies guidelines. The search strategy was initially created for PubMed and used key search criteria specific to that database. It was then modified to suit subsequent searches across other databases. The detailed search strategy is provided in Tables 2 and 3.

**Table 2.** Search strategy for brain natriuretic peptide (BNP) and aortic regurgitation.

Database and search number		Query used	Results
PubMed^a^			
	#1	Aortic regurgitation	31,031
	#2	Mixed aortic valve disease	782
	#3	#1 OR #2	31,394
	#4	BNP	13,846
	#5	Brain natriuretic peptide	24,456
	#6	#4 OR #5	28,646
	#7	#3 AND #6	138
Embase^a^			
	#1	Aortic regurgitation	57,763
	#2	Mixed aortic valve disease	1272
	#3	#1 OR #2	58,275
	#4	BNP	31,497
	#5	Brain natriuretic peptide	73,970
	#6	#4 OR #5	85,944
	#7	#3 AND #6	1603
Cochrane Library^b^			
	#1	Aortic regurgitation	716
	#2	Mixed aortic valve disease	61
	#3	#1 OR #2	762
	#4	BNP	6664
	#5	Brain natriuretic peptide	3773
	#6	#4 OR #5	8046
	#7	#3 AND #6	25

a Limit criteria: inception to November 28, 2024.

b Limit criteria: title, abstract, and keyword; inception to November 28, 2024.

**Table 3.** Search strategy for global longitudinal strain (GLS) and aortic regurgitation.

Database and search number		Query used	Results
PubMed^a^			
	#1	Aortic regurgitation	31,031
	#2	Mixed aortic valve disease	782
	#3	#1 OR #2	31,394
	#4	Global Longitudinal Strain	7342
	#5	GLS	5858
	#6	#4 OR #5	9861
	#7	#3 AND #6	114
Embase^a^			
	#1	Aortic regurgitation	57,763
	#2	Mixed aortic valve disease	1272
	#3	#1 OR #2	58,275
	#4	Global Longitudinal Strain	14,412
	#5	GLS	12,609
	#6	#4 OR #5	18,851
	#7	#3 AND #6	438
Cochrane Library^b^			
	#1	Aortic regurgitation	716
	#2	Mixed aortic valve disease	61
	#3	#1 OR #2	762
	#4	Global Longitudinal Strain	828
	#5	GLS	618
	#6	#4 OR #5	1052
	#7	#3 AND #6	4

a Limit criteria: inception to November 28, 2024.

b Limit criteria: title, abstract, and keyword; inception to November 28, 2024.

### Screening

**Figure 1.**
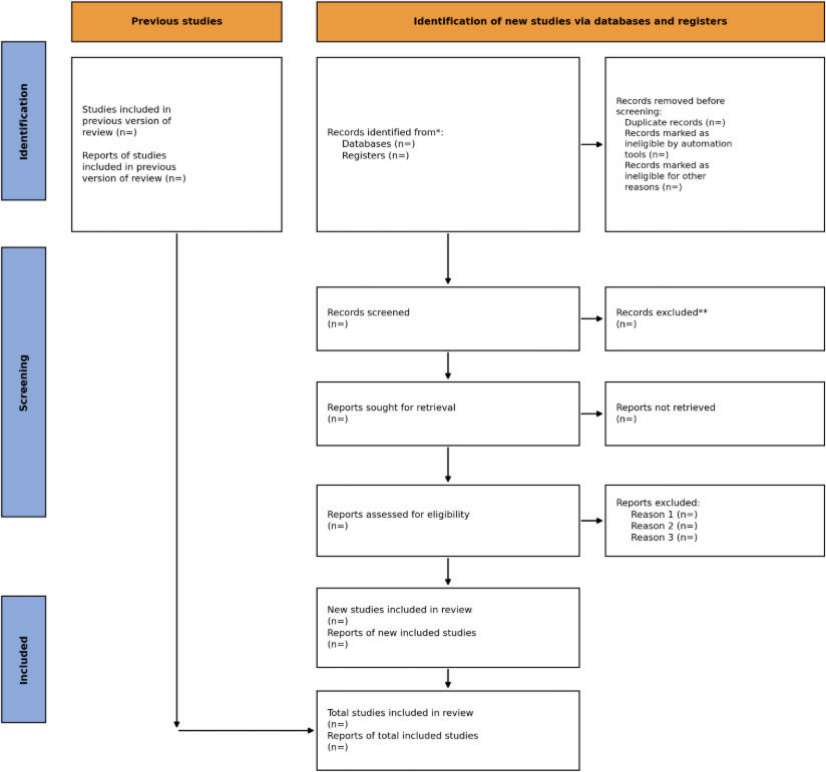
The PRISMA (Preferred Reporting Items for Systematic Reviews and Meta-Analyses) flow diagram illustrating the study selection process (reproduced from Rethlefsen et al [24]).

Every study identified using the aforementioned search strategy was reviewed by ≥4 independent researchers to ensure that every study met the inclusion criteria. This was done in a systematic manner, including reviews of the title, abstract, and full text. A PRISMA (Preferred Reporting Items for Systematic Reviews and Meta-Analyses) flow diagram (Figure 1 [24]) will be included in the final review to further detail the screening process.

### Data Extraction and Main Data Elements

Four research team members individually extracted data from studies deemed eligible through the screening process to ensure that a thorough and accurate review was performed. Differences in findings following data extraction were discussed with the first author and another independent team member to determine the appropriate use. In addition to the outcomes listed below, the following data will be extracted from each eligible study: (1) study characteristics, including first author, publication year, study design, sample size, and follow-up duration; (2) patient characteristics, including mean age, sex distribution, and relevant comorbidities; and (3) biomarker data, including mean or median BNP or N-terminal pro-B-type natriuretic peptide values, predefined thresholds used, and method of BNP measurement; and, for GLS, the echocardiography vendor and speckle-tracking software used.

The outcomes of the meta-analysis or systematic review included: (1) symptom development (frequency, timing, and severity); (2) changes in LV morphology and function (LV EF; LV systolic and diastolic diameters); (3) all-cause mortality; (4) hospitalizations (initial hospitalizations, rehospitalizations, and length of hospital stay); (5) the need for intervention based on disease progression, including transcatheter or surgical aortic valve replacement (with interventions for other indications excluded; eg, multivessel coronary artery disease requiring coronary artery bypass grafting with concomitant aortic valve replacement); and (6) MACEs.

### Quality or Risk of Bias Assessment

All studies that met the inclusion criteria and were included in the final publications were assessed using the National Heart, Lung, and Blood Institute quality assessment tool [25]. Evaluation was performed using the National Heart, Lung, and Blood Institute criteria detailed in (Textbox 1). In the event that any RCTs are identified and included, the Cochrane Risk of Bias 2 tool will be applied to assess their methodological quality, as this instrument is specifically designed for evaluating randomized study designs.

Textbox 1. **National Heart, Lung, and Blood Institute scoring checklist.**

**Quality assessment of controlled intervention studies**

1. Was the study described as randomized, a randomized trial, a randomized clinical trial, or an RCT?
2. Was the method of randomization adequate (ie, use of randomly generated assignment)?
3. Was the treatment allocation concealed (so that assignments could not be predicted)?
4. Were study participants and providers blinded to treatment group assignment?
5. Were the people assessing the outcomes blinded to the participants' group assignments?
6. Were the groups similar at baseline on important characteristics that could affect outcomes (eg, demographics, risk factors, co-morbid conditions)?
7. Was the overall drop-out rate from the study at endpoint 20% or lower of the number allocated to treatment?
8. Was the differential drop-out rate (between treatment groups) at endpoint 15 percentage points or lower?
9. Was there high adherence to the intervention protocols for each treatment group?
10. Were other interventions avoided or similar in the groups (eg, similar background treatments)?
11. Were outcomes assessed using valid and reliable measures, implemented consistently across all study participants?
12. Did the authors report that the sample size was sufficiently large to be able to detect a difference in the main outcome between groups with at least 80% power?
13. Were outcomes reported or subgroups analyzed prespecified (ie, identified before analyses were conducted)?
14. Were all randomized participants analyzed in the group to which they were originally assigned, ie, did they use an intention-to-treat analysis?

### Strategy for Meta-Analysis

The statistical analysis for this meta-analysis will include several key steps to assess the prognostic value of BNP and GLS in patients with severe asymptomatic AR and preserved EF. First, data will be pooled using a random-effects model, as this model accounts for potential variability between studies [26]. For continuous outcomes, such as changes in LV morphology and function (eg, LV EF, systolic and diastolic diameters), mean differences or standardized mean differences will be calculated. For dichotomous outcomes, such as symptom development, hospitalization rates, and the occurrence of MACEs, risk ratios or odds ratios with 95% CIs will be computed. The outcomes of interest include symptom development (frequency, timing, and severity), changes in LV function, all-cause mortality, hospitalizations (initial hospitalizations, rehospitalizations, and length of stay), the need for intervention based on disease progression (eg, transcatheter or surgical aortic valve replacement), and MACEs. Subgroup analyses will be conducted to explore potential differences based on factors such as BNP and GLS thresholds, age, sex, and study design (RCTs vs observational studies). Sensitivity analyses will be performed by excluding studies with a high risk of bias or small sample sizes to evaluate the robustness of the results. The heterogeneity of the results will be assessed using the *I*^2^ statistic, and if significant heterogeneity is detected, meta-regression and subgroup analyses will be used to explore its sources. Publication bias will be assessed using funnel plots and the Egger test [27]. Statistical significance will be defined as a 2-sided *P* value of <.05. The Comprehensive Meta-Analysis package (version 4.0; Biostat Inc) will be used to analyze the data.

### Data Analysis and Presentation

Assuming sufficient data are extracted, the data will be displayed as a final table, accompanied by a summary detailing the final analysis performed. A data extraction form will be created and reviewed by all investigators prior to analysis. The articles that are part of the final publication will constitute the horizontal rows within the table, and the multiple variables identified by the team will serve as the columns. Quantitative analysis will be performed on all variables extracted from the available data.

## Results

Search strategy development for this systematic review began in November 2024. A Peer Review of Electronic Search Strategies review of the search strategy with 2 academic librarians occurred in December 2024, and the final search strategy was finalized by the team of investigators in January 2025. Database queries and screening of studies began in January 2025 with title screening, followed by abstract screening in January 2025 and February 2025. Full-text screening will take place from February 2025 to April 2025. Data extraction will occur between April 2025 and May 2025.

Synthesis and risk of bias assessment will occur between April 2026 and May 2026, followed by data analysis between June 2026 and July 2026. Manuscript drafting will begin between June 2026 and July 2026**,** with manuscript writing and data dissemination continuing from May 2026 to August 2026. Findings will be submitted to a peer-reviewed journal by August 2026. The overall goal of this effort is to determine the utility of GLS and BNP as predictive prognostic biomarkers for asymptomatic severe AR with preserved EF. A timeline is presented in Table 4.

**Table 4.** Project milestones and timelines.

Tasks	November 2024	December 2024	January 2025	February 2025	March 2025	April 2025	May 2025	April 2026	May 2026	June 2026	July 2026	August 2026
Protocol development	✓											
Search strategy development		✓	✓									
Title screening			✓									
Abstract screening			✓	✓								
Full-text screening				✓	✓	✓						
Data extraction						✓	✓					
Synthesis and risk of bias assessment								✓	✓			
Data analysis										✓	✓	
Manuscript drafting										✓	✓	
Manuscript submission and peer review												✓

## Discussion

### Overview

The purpose of this meta-analysis is to determine the prognostic value of GLS and BNP in patients with asymptomatic severe AR and preserved EF. We will analyze the correlation of these 2 biomarkers with disease progression by examining symptom development and changes in LV function, and by identifying any additional indications requiring surgical intervention. We will also assess MACEs, including myocardial infarction, coronary revascularization, stroke, hospitalization due to heart failure, and all-cause mortality, to estimate the association of these 2 biomarkers with clinical outcomes. The results of our meta-analysis will be valuable in assessing these 2 biomarkers as independent variables for risk stratification and the need for early surgical intervention in these patient populations. This will improve prognosis and ultimately enhance the quality of life in this patient group.

### Significance

By analyzing all the existing clinical trials conducted to date, our study will solidify the current evidence regarding the prognostic value of BNP and GLS in patients with asymptomatic severe AR and preserved EF. There is no prior meta-analysis or systematic review analyzing BNP as a prognostic marker in these patient populations, indicating the need for novel analysis. GLS has been researched before; however, the most recent meta-analysis published in 2024 combined both AR and aortic stenosis. The meta-analysis published in 2020 focused only on AR; however, given new research, it is now out of date. Our meta-analysis will include primary RCTs and secondary, or follow-up, RCTs derived from the original studies. This will extend the end points available for subgroup analysis and broaden our understanding of these 2 biomarkers in this population.

Additionally, this study will guide future research by improving the knowledge regarding the potential of BNP and GLS in patients with asymptomatic severe AR and preserved EF. If enough sample studies are available for pooling, our review will be the largest meta-analysis with quantitative analysis on this topic.

### Limitations

Our meta-analysis will have several limitations. Heterogeneity will exist due to the individually selected studies, as each study has its own methodologies, settings, primary and secondary end points, time periods, and length of follow-up used to track the biomarkers. Additionally, there is an inherent risk of reviewer bias while filtering articles during the literature search. This will be mitigated by using individualized searching processes with 2 or 3 independent reviewers for each biomarker, resulting in a total of 5 independent reviewers for the 2 biomarkers, and subject matter experts will monitor every step of this meta-analysis. We will assess the risk of bias in the selected studies with standardized tools. Finally, there is a risk of missing literature applicable to our research topic.

### Dissemination Plan

The findings from this meta-analysis will be disseminated through a manuscript submitted to a peer-reviewed journal by August 2026, and the abstract originating from the manuscript will be submitted and presented at academic conferences.

### Conclusions

This study is a comprehensive review of GLS and BNP as prognostic markers in patients with asymptomatic severe AR and preserved EF. The primary end point will be symptom development, changes in LV function, or the emergence of additional indications requiring surgical intervention. The secondary end points will include hospitalizations (initial hospitalizations, rehospitalizations, and length of hospital stay), MACEs, and all-cause mortality. Overall, by aggregating all the available literature relevant to our topic, we anticipate that our analysis will evaluate the reliability of these 2 biomarkers for predicting the prognosis in this patient population, and we hope that this study may provide insights into future directions for improving health outcomes in this population.

## Supplementary material

10.2196/71380 Checklist 1 PRISMA (Preferred Reporting Items for Systematic Reviews and Meta-Analyses) checklist.

## Abbreviations

AR: aortic regurgitation
BNP: brain natriuretic peptide
GLS: global longitudinal strain
LV: left ventricular
MACE: major adverse cardiac event
PRISMA: Preferred Reporting Items for Systematic Reviews and Meta-Analyses
RCT: randomized controlled trial
